# The Murine Stem Cell Virus Promoter Drives Correlated Transgene Expression in the Leukocytes and Cerebellar Purkinje Cells of Transgenic Mice

**DOI:** 10.1371/journal.pone.0051015

**Published:** 2012-11-30

**Authors:** Miho Oue, Hiroshi Handa, Yasunori Matsuzaki, Kazutomo Suzue, Hirokazu Murakami, Hirokazu Hirai

**Affiliations:** 1 Department of Neurophysiology, Gunma University Graduate School of Medicine, Maebashi, Gunma, Japan; 2 Department of Medicine and Clinical Science, Gunma University Graduate School of Medicine, Maebashi, Gunma, Japan; 3 Department of Parasitology, Gunma University Graduate School of Medicine, Maebashi, Gunma, Japan; 4 Department of Laboratory Sciences, Gunma University Graduate School of Health Sciences, Maebashi, Gunma, Japan; University of Pittsburgh School of Medicine, United States of America

## Abstract

The murine stem cell virus (MSCV) promoter exhibits activity in mouse hematopoietic cells and embryonic stem cells. We generated transgenic mice that expressed enhanced green fluorescent protein (GFP) under the control of the MSCV promoter. We obtained 12 transgenic founder mice through 2 independent experiments and found that the bodies of 9 of the founder neonates emitted different levels of GFP fluorescence. Flow cytometric analysis of circulating leukocytes revealed that the frequency of GFP-labeled leukocytes among white blood cells ranged from 1.6% to 47.5% across the 12 transgenic mice. The bodies of 9 founder transgenic mice showed various levels of GFP expression. GFP fluorescence was consistently observed in the cerebellum, with faint or almost no fluorescence in other brain regions. In the cerebellum, 10 founders exhibited GFP expression in Purkinje cells at frequencies of 3% to 76%. Of these, 4 mice showed Purkinje cell-specific expression, while 4 and 2 mice expressed GFP in the Bergmann glia and endothelial cells, respectively. The intensity of the GFP fluorescence in the body was relative to the proportion of GFP-positive leukocytes. Moreover, the frequency of the GFP-expressing leukocytes was significantly correlated with the frequency of GFP-expressing Purkinje cells. These results suggest that the MSCV promoter is useful for preferentially expressing a transgene in Purkinje cells. In addition, the proportion of transduced leukocytes in the peripheral circulation reflects the expression level of the transgene in Purkinje cells, which can be used as a way to monitor transgene expression properties in the cerebellum without invasive techniques.

## Introduction

The Moloney murine leukemia virus (MoMLV)-based retrovirus vector has been widely used to transfer genes into dividing eukaryotic cells [1]. MoMLV and MoMLV-derived retroviral vectors are not active in undifferentiated mouse embryonic stem cells or in embryonic carcinoma cells due to several inhibitory mechanisms, including DNA methylation, a lack of enhancer function and the presence of negative transacting factors that result in the subsequent transcriptional silencing of the 5′ long terminal repeat (LTR) promoter region [2]–[6]. A newer-generation murine stem cell virus (MSCV) vector was developed from the MoMLV vectors. The upstream region of the LTR in the MSCV vector was replaced with the homologous region from the Moloney murine sarcoma virus [7], [8], which differs from the MoMLV LTR by several point mutations and a deletion. These changes allow the MSCV vector to influence transcriptional activity in embryonic stem cells and in embryonic carcinoma cells. The MSCV promoter, which consists of the 5′ LTR and the packaging signal, ψ^+^, from the MSCV vector, has previously been used for the transduction of hematopoietic and embryonic stem cells [9]–[15].

We previously demonstrated that cerebellar injection of lentiviral vectors expressing enhanced green fluorescent protein (GFP) under the control of the MSCV promoter led to the transduction of various types of neuronal and glial cerebellar cells, and that the highest transduction efficiency was observed in Purkinje cells [16], [17]. Moreover, the MSCV promoter transduced Purkinje cells more efficiently than other viral promoters, such as the cytomegalovirus (CMV) promoter, the CMV early enhancer/chicken β actin (CAG) promoter and the Rous sarcoma virus (RSV) promoter [18]. However, the cell types that are transduced by the vectors largely depend on the infectious tropism of the viral vectors. For example, lentiviral vectors expressing a transgene under the control of the MSCV promoter primarily transduced Bergmann glia when the viruses were exposed to low pH [17], when the viruses were harvested after prolonged cultivation [19], or when a different serum lot was used to supplement the culture medium (Protocol Exchange, 2007, doi:10.1038/nprot.2007.89). Thus, our previous study [18] indicates that the MSCV promoter preferentially transduces Purkinje cells in combination with Purkinje cell-tropic lentiviral vectors. The specificity of the MSCV promoter in Purkinje cells, or in other cell types in the cerebellum and other brain regions, has not been verified.

To examine MSCV promoter activity in the brain, we generated transgenic mice that expressed GFP under the control of the MSCV promoter. We found that the transgenic mice preferentially expressed GFP in Purkinje cells and in circulating hematopoietic cells, whereas other brain areas expressed faint or no GFP expression. Interestingly, the ratio of GFP-expressing Purkinje cells to all Purkinje cells in the cerebellum was significantly correlated with that of GFP-expressing leukocytes.

## Results

### Ubiquitous Gene Expression Under the Control of the MSCV Promoter in Cultured Cells

**Figure 1:**
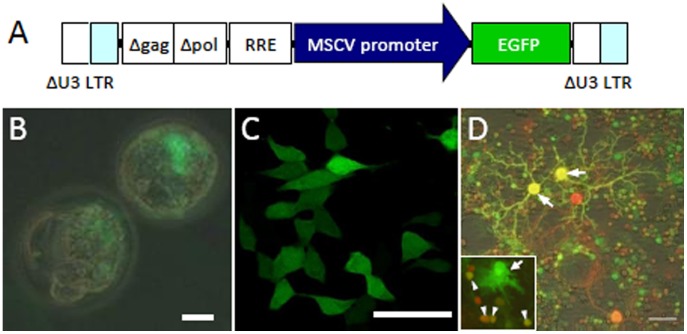
MSCV promoter-mediated expression of GFP in various types of cultured cells. (A) Lentiviral vectors express enhanced green fluorescent protein (GFP) under the control of the MSCV promoter. To remove TAT-dependent transcription, the U3 region of the LTR was deleted. The Δgag (deleted gag sequence) was included to enhance packaging efficiency. For improved viral-mediated gene transfer, the central polypurine tract (cPPT) and central termination sequence (CTS) were added in Δpol (deleted pol sequence). RRE; Rev response element. (B) Expression of GFP in mouse embryos. (C) Expression of GFP in human embryonic kidney (HEK) 293T cells. (D) Cerebellar neuronal culture infected with lentiviral vectors at 1 day in vitro (DIV) and immunolabeled for neuron-specific nuclear protein (NeuN) at DIV 15. In addition to Purkinje cells (arrows), GFP was expressed in granule neurons (arrowhead, inset) and in glia (arrow, inset). Scale bars, 20 µm (B), 25 µm (C), 50 µm (D).

Lentiviral vectors expressing GFP under the control of the MSCV promoter (Fig. 1A) were used to infect two-cell embryos. Three days after infection, GFP expression in the embryos was observed (Fig. 1B) and was consistent with a previous report showing MSCV promoter activity in embryonic stem cells [7]. The MSCV promoter was also active in HEK 293T cells (Fig. 1C) and in various types of cultured cerebellar cells (Fig. 1D), including Purkinje cells (arrow), granule cells (arrowhead, inset in Fig. 1D) and glia (arrow, inset in Fig. 1D). The types of cerebellar cells that expressed GFP were confirmed using antibodies specific to each cell type (data not shown). These results suggest that the MSCV promoter is ubiquitously active in cultured cells.

### Generation of Transgenic Mice Using Lentiviral Vectors

**Figure 2:**
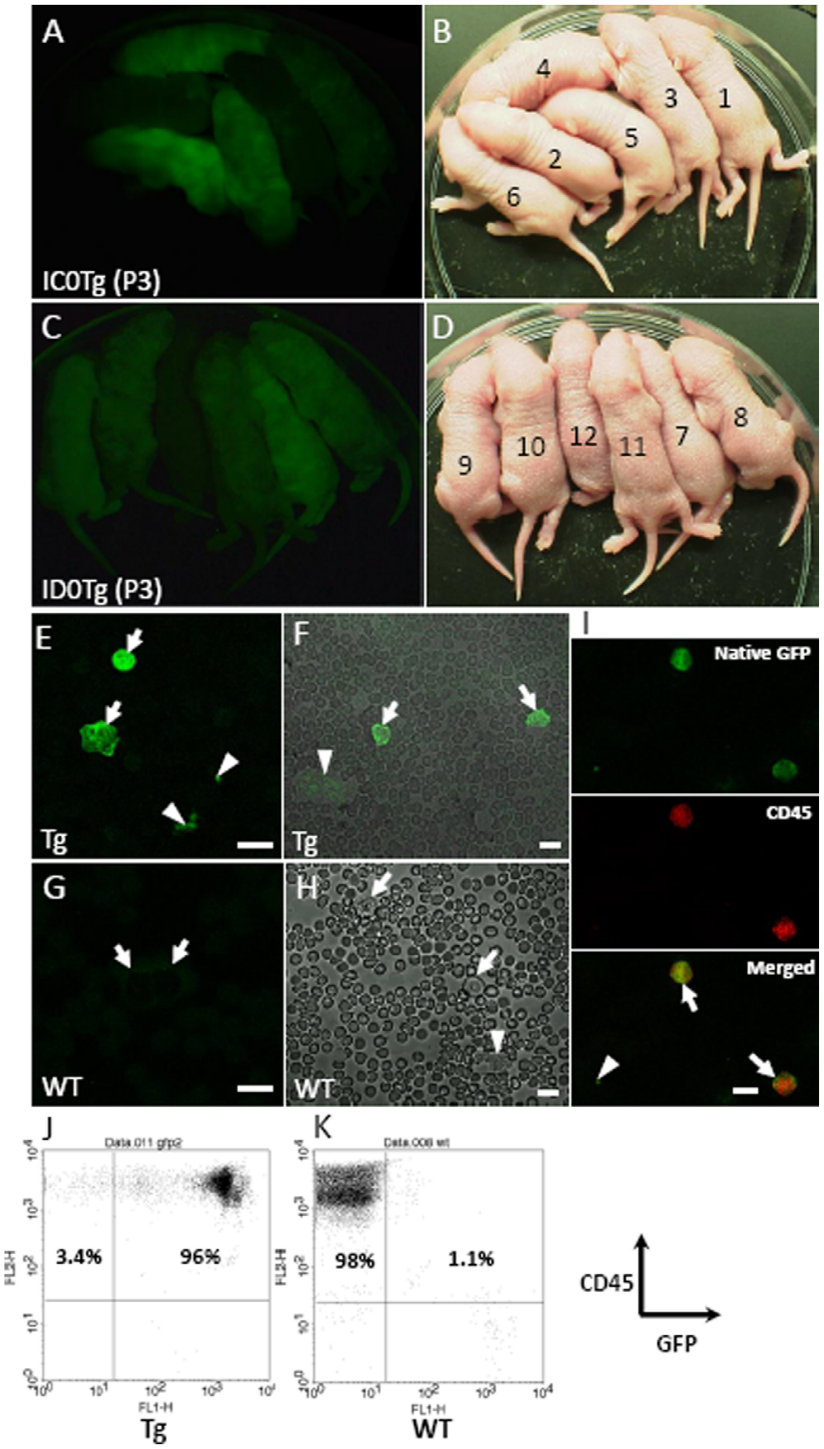
GFP expression in the bodies and leukocytes of MSCV-GFP founder mice. (A–D) Twelve founder pups were obtained from 2 independent experiments (A, B) and (C, D). Brightfield (B, D) and GFP fluorescence images (A, C) of the transgenic pups were obtained using a fluorescence stereoscopic microscope. (E–H) Robust GFP expression was observed in the leukocytes (arrow) and platelets (arrowhead) of a representative MSCV-GFP mouse (ID0Tg-7, see Table 1) (E, F) but not in wild-type mice (G, H). (I) Immunolabeling of GFP-positive leukocytes (arrow) but not GFP-positive platelet (arrowhead) for CD45. Peripheral blood cells from a F1 MSCV-GFP mouse (IE1Tg-2, see Table 1) were immunolabeled for CD45. (J, K) Flow cytometric analysis of leukocytes in the MSCV-GFP mouse. Peripheral blood cells from a F1 transgenic mouse (IE1Tg-2) and a wild-type mouse were immunolabeled for CD45 following hemolysis of erythrocytes. Leukocytes were analyzed using a flow cytometer and CellQuest pro software. Scale bar, 10 µm.

Lentiviral vectors were used to introduce a cassette containing the MSCV promoter plus GFP into the genomes of ICR mouse embryos. The lentiviral vector-infected embryos were then implanted into the oviducts of pseudo-pregnant females. We conducted 2 independent experiments using 2 females and 2 batches of lentiviral vectors at titers of 5.6×10^10^ and 6.2×10^10^ transducing units/ml. The embryos were transplanted into 2 female mice (17 and 20 embryos), and a total of 12 pups were delivered (6 pups each). The results of genotyping the pups showed that all the pups carried the transgene, and hereafter, the pups carrying the transgene will be referred to as MSCV-GFP mice (Table 1). Since there were no wild-type littermates, we purchased 3 wild-type ICR mice as control animals (Table 1) from a same supplier (Japan SLC, Shizuoka Japan). The bodies of 9 out of the 12 MSCV-GFP founder neonates displayed various levels of GFP expression when viewed under a fluorescence stereoscopic microscope (Fig. 2A–D and Table 1).

**Table 1:** Properties of MSCV-GFP founder and F1 mice in comparison to wild-type mice.

ID	F/M	Body Fluorescence	FACS (% gated)	GFP(+) PC(%)	Generation	Genotype
IAWt-1	F	–	0.3	0		Wt
IAWt-2	F	–	0.3	0		
IBWt-3	F	–	1.2	0		
IC0Tg-1	M	+	12.3	21	F0	Tg
IC0Tg-2	M	–	2.8	3		
IC0Tg-3	F	–	1.6	0		
IC0Tg-4	F	+++	35.0	58		
IC0Tg-5	M	++	29.6	11		
IC0Tg-6	M	+++	33.9	76		
ID0Tg-7	F	+++	27.3	71		
ID0Tg-8	M	++	19.4	28		
ID0Tg-9	M	++	47.5	65		
ID0Tg-10	M	+	19.7	14		
ID0Tg-11	F	+	14.1	24		
ID0Tg-12	M	–	1.8	0		
IE1Tg-1	F	+++	92.2		F1(IC0Tg-6×Wt)	Tg
IE1Tg-2	F	+++	92.0			

The first letter (I) of the identification (ID) indicates that the mouse was generated on an ICR background. Littermates have the same letter (A–E) at the second position. Numbers (0 or 1) at the third position show the filial generation of the transgenic mice and are followed by an abbreviation of the genotype, either wild-type (Wt) or transgenic (Tg). The final numbers are serial numbers in the same filial generation. Since there was no wild type in the F0 generation and also in the F1 offspring, the wild-type ICR mice (IAWt-1, IAWt-2 and IBWt-3) were obtained from a same supplier. F; female, M.

### GFP Expression in Hematopoietic Cells of MSCV-GFP Mice

Because retroviral and lentiviral vectors containing the MSCV promoter have often been used for gene transfer into cells from hematopoietic lineages [9], [11], [12], [14], [15], we examined GFP expression in the peripheral blood cells of the MSCV-GFP mice. Using a fluorescence microscope, we observed GFP expression in the leukocytes of the MSCV-GFP mice (Fig. 2E–F, arrow) but not in those of wild-type mice (Fig. 2G, H), arrow). No GFP fluorescence was detected in the erythrocytes of the MSCV-GFP mice. Additionally, we observed GFP fluorescence in platelets of the MSCV-GFP mouse but not in those of wild-type mice (Fig. 2E, F, H, arrowhead). To confirm GFP expression in the leukocytes, cells in the peripheral blood of a F1 transgenic mouse (IE1Tg-2, see Table 1) and a wild-type mouse were immunolabeled for CD45 (leukocyte common antigen, Ly-5). GFP-expressing leukocytes but not a platelet were immunolabeled for CD45 (Fig. 2I). Leukocyte GFP expression was also examined by flow cytometry (FACSCalibur; Becton Dickinson, San Jose, CA, USA) after immunolabeling of leukocytes with the antibody to CD45. GFP was expressed in 96% of the CD45-labeled leukocytes in the F1 MSCV-GFP mouse (Fig. 2J), whereas GFP was expressed only at background levels (1.1%) in the CD45-labeled leukocytes from the wild-type mouse (Fig. 2K).

Next, we examined the ratio of GFP-expressing leukocytes to the total number of leukocytes in the peripheral blood of 12 transgenic founders by flow cytometry. The mean percentage of GFP-positive leukocytes was 20.4±4.2% (n = 12 animals) and ranged from 1.6 to 47.5%, while the background values from 3 wild-type ICR mice were 0.3, 0.3 and 1.2% (Table 1).

### Preferential Expression of GFP in the Purkinje Cells of MSCV-GFP Mice

We examined transgene expression in the brains of 12 MSCV-GFP founder mice. Almost no GFP fluorescence was observed in 6 founder mice under the fluorescence stereoscopic microscope. The remaining 6 founder mice showed different degrees of GFP expression in the cerebella, with faint or almost no GFP expression in other brain regions. Next, we examined the GFP-expressing cell types in the cerebellum in sagittal sections from 12 founders. GFP-expressing cells were identified in the cerebella of 10 mice, all of which displayed various levels of GFP expression in the Purkinje cells, although the proportions of the number of transduced Purkinje cells to the total number of Purkinje cells differed greatly (Fig. S1A). The mean percentage of GFP-expressing Purkinje cells was 30.9±8.3% (n = 12 founder mice) and ranged from 0 to 76% (Table 1). Four out of 10 founders with GFP-positive cells in the cerebellum exhibited a Purkinje cell-specific pattern of GFP expression (IC0Tg-5, ID0Tg-9, ID0Tg-10 and ID0Tg-11) (Fig. S1B), whereas, in addition to Purkinje cells, Bergmann glia (Fig. S1C) and endothelial cells (Fig. S1D) were transduced in 4 (IC0Tg-1, IC0Tg-4, IC0Tg-6 and ID0Tg-7) and 2 (IC0Tg-2, ID0Tg-8) founder mice, respectively. These results suggest that Purkinje cells are preferentially, and in some cases specifically, transduced by the MSCV promoter.

### Correlation between GFP Expression in Leukocytes and in Purkinje Cells

**Figure 3:**
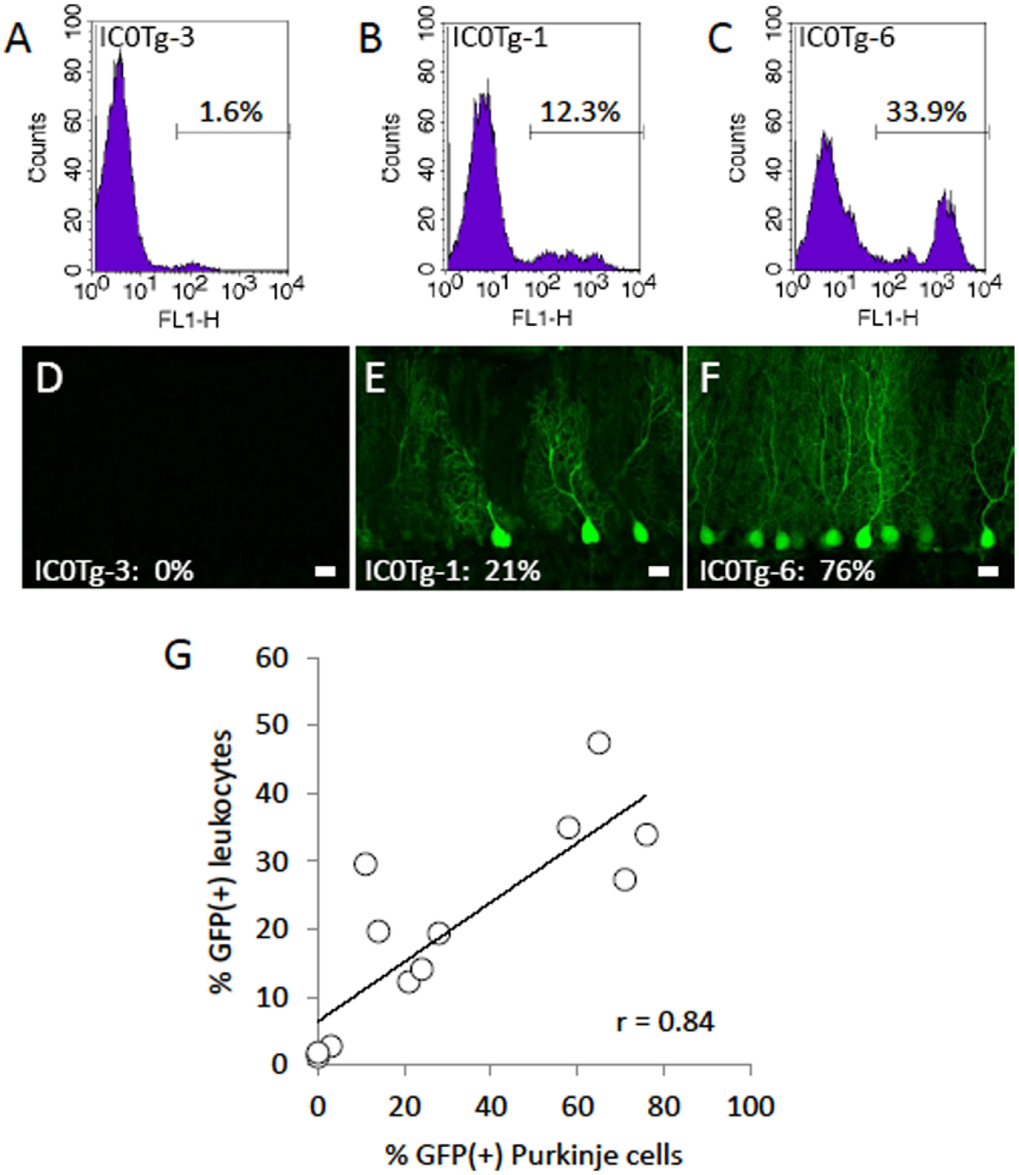
Significant correlation between the frequencies of GFP-positive leukocytes and GFP-expressing Purkinje cells. (A–C) GFP fluorescence of peripheral circulating leukocytes was analyzed by flow cytometry. (D–F) Sagittal sections of the cerebellar vermis from the same animals (A–C) are shown. The results were obtained from IC0Tg-3 (A, D), IC0Tg-1 (B, E) and IC0Tg-6 (C, F) mice (see Table 1). Scale bar, 20 µm. (G) The frequency of GFP-expressing leukocytes (% GFP(+) leukocytes) obtained from 12 MSCV-GFP founder mice was plotted against the frequency of GFP-expressing Purkinje cells (% GFP(+) Purkinje cells). The correlation coefficient (r) was 0.84, indicating a significant correlation between the two values.

We found that the strength of GFP fluorescence in the body reflected the frequency of GFP-expressing Purkinje cells, which could be used as a basic measure of the frequency of GFP-positive leukocytes (Table 1). The upper graphs in Figure 3 represent examples of the flow cytometric analyses obtained from 3 MSCV-GFP mice, in which 1.6% (IC0Tg-3) (Fig. 3A), 12.3% (IC0Tg-1) (Fig. 3B) and 33.9% (IC0Tg-6) (Fig. 3C) of circulating leukocytes expressed GFP, and 0% (Fig. 3D), 21% (Fig. 3E) and 76% (Fig. 3F) of cerebellar Purkinje cells expressed GFP. To confirm this correlation, the frequency of GFP-expressing Purkinje cells was plotted against the frequency of GFP-positive leukocytes (Fig. 3G). The correlation coefficient (*r*) was 0.84, indicating that there is significant linear dependence (*p*<0.01) between the ratio of the number of GFP-positive leukocytes to the total number of leukocytes and to the number of GFP-expressing Purkinje cells in the cerebellum of MSCV-GFP mice.

We further analyzed the correlations between the intensity of GFP fluorescence in the body and the frequency of GFP-positive leukocytes and between the intensity of GFP fluorescence in the body and the frequency of GFP-expressing Purkinje cells. In this analysis, GFP fluorescence in the body (−, +,++ and +++) was regarded as (0, 1, 2 and 3), respectively. The correlation coefficients (*r*) were 0.85 and 0.88, respectively, which indicates significant correlations between the intensity of GFP fluorescence in the body and the ratio of the number of GFP-positive leukocytes to the total number of leukocytes (*p*<0.01) and between the intensity of GFP fluorescence in the body and the ratio of the number of GFP-positive Purkinje cells to the total number of Purkinje cells (*p*<0.01).

### Generation and Analysis of MSCV-GFP Mice Using a C57BL/6 Strain

**Figure 4:**
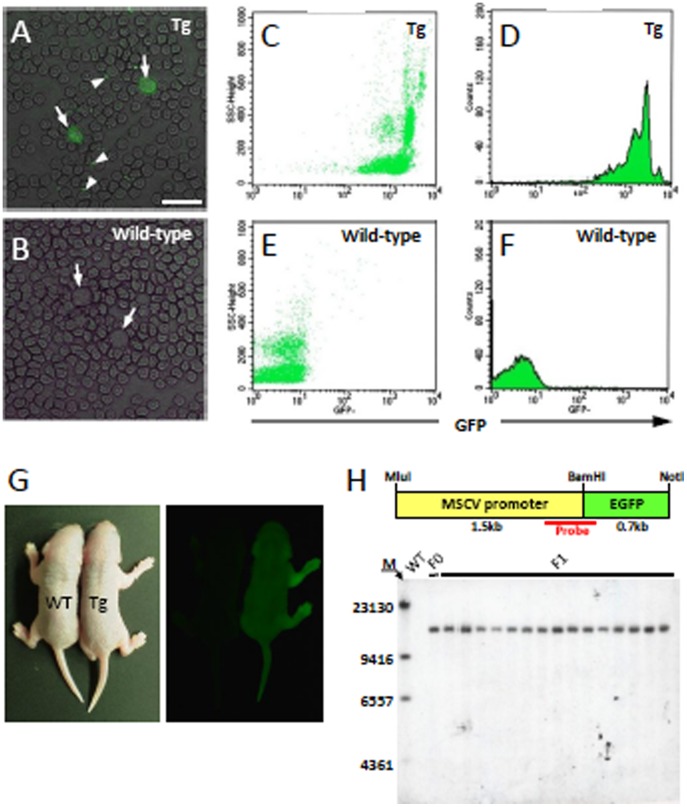
GFP expression in the body and leukocytes of a C57BL/6 MSCV-GFP mouse. (A, B) GFP expression in circulating leukocytes (arrow) and platelets (arrowhead) in a transgenic mouse (A) but not in a wild-type littermate (B). (C–F) Flow cytometric analysis shows that almost all of the circulating leukocytes in the transgenic mice (C, D) have higher GFP fluorescence levels than those of wild-type mice (E, F). (G) Illumination of a F1 transgenic mouse pup (Tg) at 3 days of age, together with a wild-type littermate (WT). (H) Southern blot analysis of the blood of the founder mouse (F0), 15 F1 pups (F1) and a wild-type littermate (WT). Upper illustration shows the genomic region (450 bp) where the DIG-labeled probe bound. The lane marker (M) on the left of the gel represents base pairs. Scale bar, 20 µm.

The Purkinje cells and leukocytes of gene-targeted mice can be labeled with GFP by crossing them with MSCV-GFP mice. The GFP-labeled mice are useful for two-photon in vivo live imaging, FACS sorting or other types of morphological analyses. Here, we first used ICR mice for the production of transgenic mice and the subsequent characterization of the MSCV promoter because ICR mice are superior to other strains in terms of the number and size of eggs that are produced and their reproductive vigor. However, because many gene-targeting mice are generated using the C57BL/6 genetic background, we tried to generate MSCV-GFP mice in the C57BL/6 background strain using a lentiviral vector-based approach. Several genotype-positive transgenic founder mice were obtained together with genotype-negative littermates, from which we selected one transgenic mouse that expressed bright GFP body fluorescence and nearly complete labeling of leukocytes with GFP (Fig. 4A–F) for further characterization. The founder male mouse was crossed to 3 wild-type C57BL/6 females and a total of 15 genotype-positive F1 mice, along with their wild-type littermates, were obtained in a Mendelian distribution. The bodies of the genotype-positive F1 mice, but not genotype-negative mice, emitted GFP fluorescence under a fluorescence stereoscopic microscope (Fig. 4G).

The number of transgene copies in the MSCV-GFP founders and in the 15 genotype-positive offspring was determined by Southern blot analysis. The genomes from these mice were digested with *Bam*HI, separated by electrophoresis and transferred to a Hybond N+ nylon membrane. Bound DNA was hybridized with a digoxigenin (DIG)-labeled probe directed to the 450-bp region containing the boundary between the MSCV promoter and GFP (Fig. 4H). The hybridized probe was detected with an alkaline phosphatase-conjugated anti-DIG antibody. Only one band was detected in all of the lanes that were loaded with samples from these mice (Fig. 4H), suggesting that the transgene was inserted at a single locus in the genome of the founder and in his transgenic progeny.

### GFP was Expressed in Almost All of the Purkinje Cells in the C57BL/6 MSCV-GFP Mice

**Figure 5:**
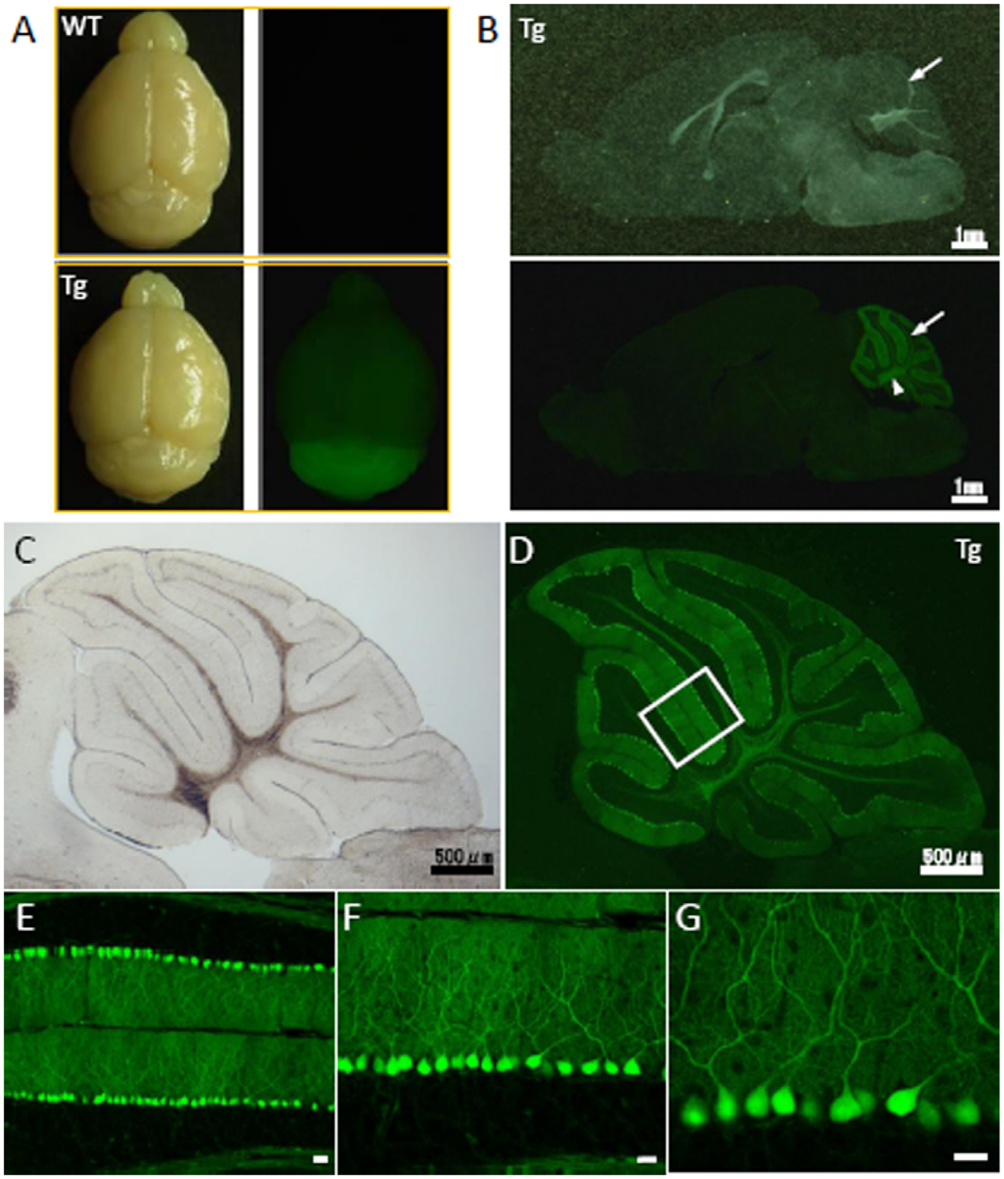
Cerebellum-restricted expression of GFP in the C57BL/6 MSCV-GFP mouse line. (A) Brightfield (left) and fluorescent (right) images of the brains of transgenic mice (lower) and their wild-type littermates (upper). A high level of GFP expression was observed in the C57BL/6 MSCV-GFP mouse cerebellum. (B) A sagittal section of a C57BL/6 MSCV-GFP mouse brain is shown, again showing the cerebellum-selective expression of GFP. Scale bar, 1 mm. (C–G) Purkinje cell-specific expression of GFP in the C57BL/6 MSCV-GFP mouse cerebellum shown by brightfield (C) and fluorescent images (D). The boxed area in (D) is enlarged (E–G) to show the Purkinje cell-specific expression of GFP. Scale bar, 20 µm.

We further examined transgene expression in the cerebella of C57BL/6 MSCV-GFP mice. Fluorescence stereoscopic observations of the brains of MSCV-GFP mice showed that GFP was predominately expressed in the cerebellum (Fig. 5A). Sagittal sections (Fig. 5B) revealed strong GFP expression at the surface (arrow) and in the internal regions (arrowhead) of the cerebellum.

In magnified images of the C57BL/6 MSCV-GFP mouse cerebellum, we observed high levels of GFP expression in the vast majority of Purkinje cells, although some Purkinje cells lacked GFP expression (Fig. 5C, D). GFP was present in Purkinje cell dendrites in the molecular layer and in the Purkinje cell axons that projected to the deep cerebellar nuclei. Transgene expression in the cerebellum was mainly confined to Purkinje cells (Fig. 5D–G). No GFP was expressed in Bergmann glia, endothelial cells, granule cells or other cortical interneurons.

Modest expression levels of GFP were also observed in numerous other cells in the brainstem, especially in the pontine nuclei (Fig. S2), although we did not identify the types of cells that were transduced in our preliminary immunolabeling experiments. Faint GFP expression was detected in other brain regions, such as the cerebral cortex and the hippocampus. GFP in these regions was present only in vascular endothelial cells (data not shown), as determined by the expression of factor VIII-related antigen, a marker of vascular endothelial cells.

### GFP Expression in Hematopoietic Cells and Myocytes

**Figure 6:**
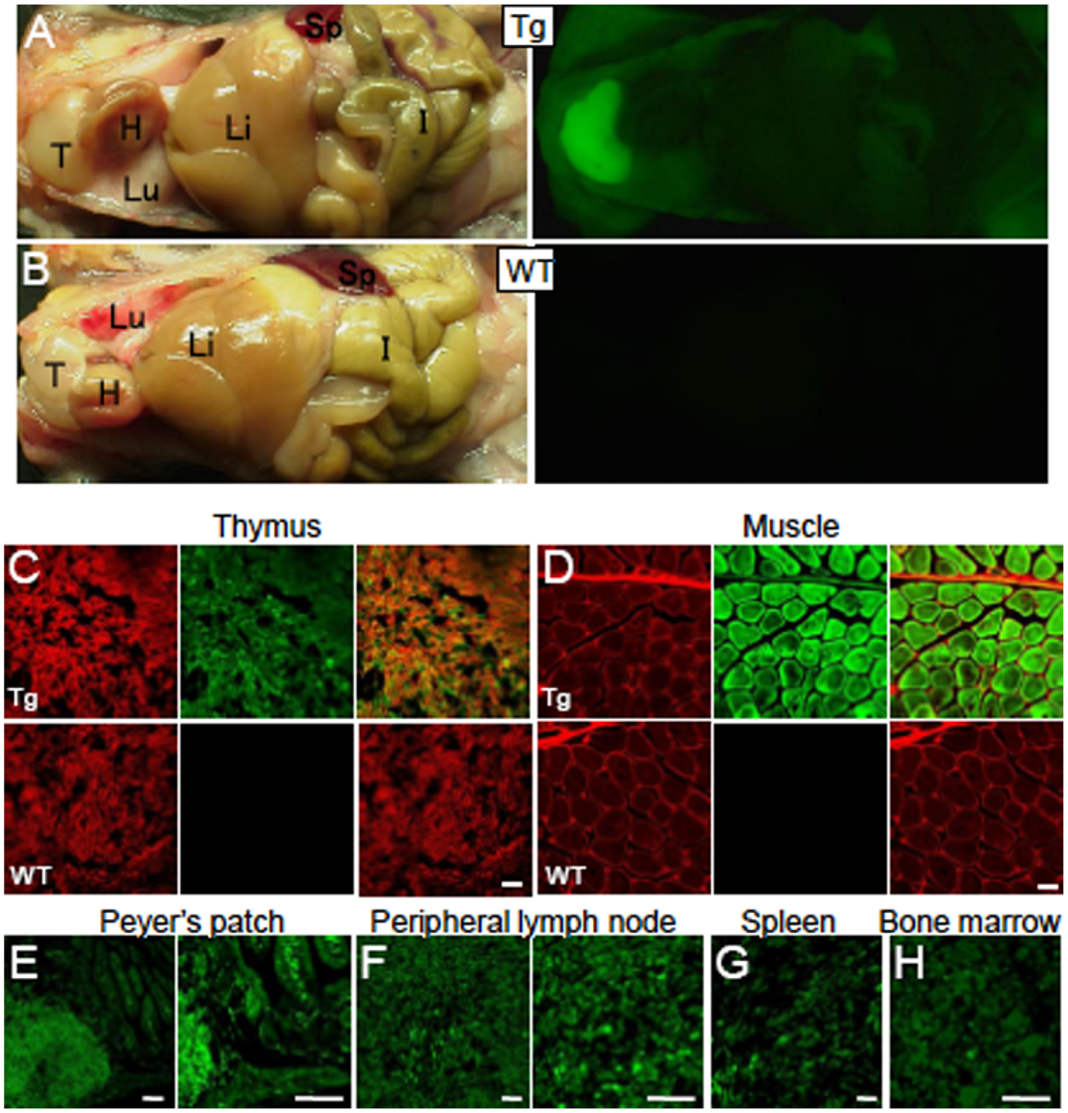
Expression of GFP in the lymphoid tissues and myocytes of C57BL/6 MSCV-GFP mice. (A, B) Brightfield (left) and fluorescent (right) images of an MSCV-GFP mouse (A) and a wild-type littermate (B). The brightest GFP fluorescence was observed in the thymus, followed by the skeletal muscle. T; thymus, H; heart, Lu; lung, Li; liver, I; intestine, Sp; spleen. (C, D) Histological sections of the thymus (C) and skeletal muscle (D) immunolabeled for GFP (green) and CD45 (C, red) or a-actinin (D, red). The upper and lower images were obtained from an MSCV-GFP mouse and a wild-type littermate, respectively. (E–H) Native GFP fluorescence images from Peyer’s patch of the intestine (E), peripheral lymph node (F), spleen (G) and bone marrow (H) of an MSCV-GFP mouse. No fluorescence signal was detected in the same regions of wild-type littermates. Scale bars, 50 µm (C), 20 µm (D), 100 µm (E), 50 µm (F) and 20 µm (G, H).

We next examined MSCV promoter activity in various tissues from the C57BL/6 MSCV-GFP mice. Fluorescent stereoscopic examination of the internal organs of P30 MSCV-GFP mice showed GFP expression in several tissues. The highest expression levels were observed in the thymus, followed by muscle and intestine (Fig. 6A, upper panels), whereas the internal organs of wild-type littermates did not express GFP (Fig. 6B, lower panels), indicating that the fluorescence observed in the MSCV-GFP mice was not intrinsic.

We examined GFP expression in histological sections of the tissues that were produced following perfusion with 4% paraformaldehyde. GFP expression in the thymus, muscle, Peyer’s patch of the intestine, peripheral lymph nodes, spleen, bone marrow and lung was confirmed (Fig. 6C–H and data not shown), whereas no GFP signals were detected in the retina, liver (hepatocyte), heart (cardiac muscle) and skin (data not shown). These results suggest that GFP was predominantly expressed in cells of hematopoietic lineage and in myocytes.

### GFP Expression during Development in C57BL/6 MSCV-GFP Mice

**Figure 7:**
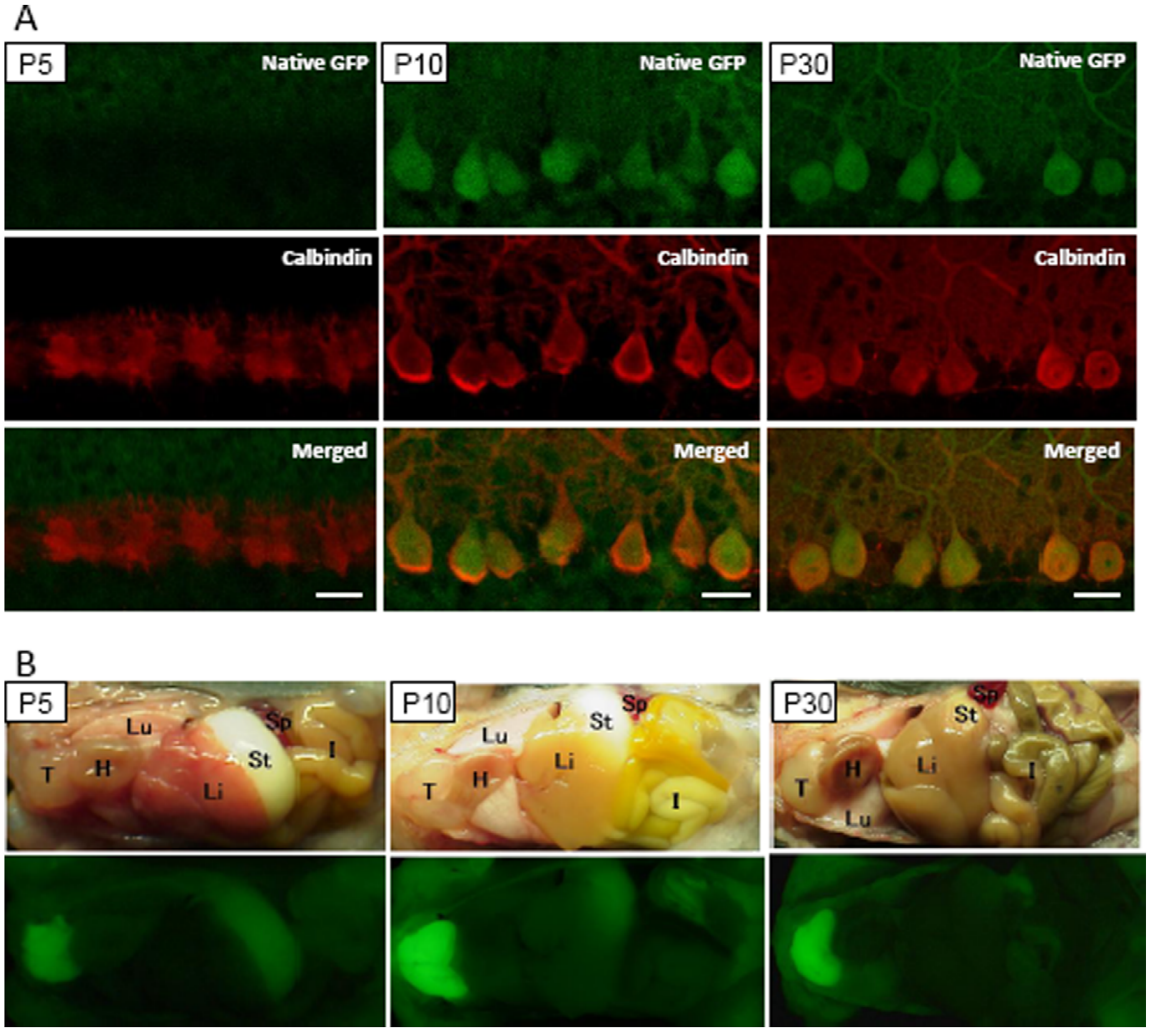
Expression of GFP during the development of C57BL/6 MSCV-GFP mice. (A) No GFP expression was observed in the Purkinje cells at P5. Cerebellar sections obtained from MSCV-GFP mice at P5 (left), P10 (middle) and P30 (right) were immunolabeled for calbindin. Images of native GFP fluorescence (upper), calbindin immunoreactivity (middle) and merged images (lower) are presented. (B) Brightfield (upper) and fluorescent (lower) images of MSCV-GFP mice at P5, P10 and P30. Weak, but clearly detectable, levels of GFP were observed in the thymus from as early as P5 and increased thereafter. GFP fluorescence in the skeletal muscles became overt at P10. T; thymus, H; heart, Lu; lung, Li; liver, I; intestine, St; stomach, Sp; spleen.

We examined GFP expression in the Purkinje cells and extra-CNS tissues of MSCV-GFP mice at P5 and P10 and compared the results to GFP expression in a P30 MSCV-GFP mouse (Fig. 7). Cerebellar sections immunolabeled for calbindin showed no GFP expression in Purkinje cells at P5, while GFP expression was clearly observed at P10, a time when the levels were almost similar to those observed at P30 (Fig. 7A). In contrast, GFP fluorescence was detectable as early as P5 in the thymus and became brighter at P10 (Fig. 7B). Apparent GFP fluorescence in the skeletal muscle was observed beginning at P10 (Fig. 7B).

## Discussion

In this study, we showed that the MSCV promoter could consistently drive transgenic GFP expression in hematopoietic cells and in Purkinje cells. Under a fluorescence stereoscopic microscope, the bodies of the transgenic mice emitted GFP fluorescence and the strength of the signal was proportional to the number of GFP-labeled leukocytes. Moreover, the frequency of GFP-labeled leukocytes correlated significantly with the frequency of GFP-expressing Purkinje cells in the cerebellum. In this study, we obtained 12 MSCV-GFP founder mice on an ICR genetic background. Of these, 2 mice did not express GFP in Purkinje cells, while the remaining 10 mice exhibited clearly detectable levels of transgene expression in Purkinje cells, although the frequency of GFP expression in the Purkinje cells varied from 3% to 76% (Fig. S1A, Table 1).

For evaluation of the MSCV-GFP ICR mice, we used commercially obtained wild-type mice as a control because there was no wild type in the F0 generation and also in the F1 offspring. Non-littermates are not animals best appropriate for control. However, in our study, there were 12 founder mice that express quite different levels of the transgene in leukocytes and cerebellar Purkinje cells, and wild-type animals obtained from a same supplier were used just to evaluate the background levels of the ICR strain. Under this condition, the ratio of transduced leukocytes was significantly correlated with density of transduced Purkinje cells in 12 founders examined, and therefore, we think that the conclusion of this paper was not influenced by the use of non-littermate wild type.

Expression of a transgene in Purkinje cells via lentiviral injection to the developing and matured cerebellum is highly effective and can be used, for example, to screen various mutants of a gene of interest [20]. However, the phenotypes of transgenic mice are much more stable than the phenotypes that are developed when lentiviral injection to the cerebellum is used to express a transgene. Therefore, the generation of transgenic mice could be more beneficial for detailed analysis of a gene of interest.

It is not yet known why the MSCV promoter shows selectively high activity in the Purkinje cells of transgenic mice. The MSCV promoter is active in embryonic stem cells, but remains sensitive to transcriptional silencing in other cell types [21], [22]. Therefore, it was not surprising to find that the MSCV promoter may have been extensively silenced during embryogenesis in the transgenic mouse brain. Indeed, no transgene expression was observed in the Purkinje cells of the transgenic mice at P5. Thus, it is plausible that the MSCV promoter, introduced to the genome after birth via the use of a lentivirus, is not silenced in most types of cerebellar cells (Fig. 1C). Thus, the question remains as to why Purkinje cells in P10 and older MSCV-GFP mice escaped transcriptional silencing of the lentivirus-inserted MSCV promoter. Because numerous genes that initiate vigorous dendritogenesis and synaptogenesis with tens of thousands of parallel and climbing fibers active in Purkinje cells from the 2^nd^ postnatal week, one possibility is that Purkinje cells begin to produce a unique transcription factor that is resistant to silencing and that activates the MSCV promoter. As the MSCV promoter is active in most hematopoietic cells, P10 and older Purkinje cells may possess a similar transcription factor.

The L7 promoter is currently used for the Purkinje cell-selective expression of transgenes. However, when lentiviral vectors are used for transgene expression in the mature rodent cerebellum, L7 promoter activity is extremely weak; therefore, it is difficult to obtain a sufficient amount of transgene expression in infected Purkinje cells [18]. In contrast, the MSCV promoter is markedly superior to the L7 promoter in terms of promoter strength [18], [23]. With respect to cell-type specificity, the L7 promoter is functional only in Purkinje cells and retinal bipolar cells [24]. In contrast, the MSCV promoter is active in Purkinje cells and in other cell subsets in the brain and the periphery. Therefore, if ataxic behavior is observed in transgenic animals expressing a particular gene under the control of the MSCV promoter, it cannot simply be concluded that their ataxia is caused by cerebellar defects. However, the influence of transgene expression in the cerebellum can be evaluated if dissociated Purkinje cells or cerebellar slice preparations from transgenic animals are used. Unlike the L7 promoter, the cerebella of MSCV promoter-driven transgenic mice contain various proportions of non-transduced Purkinje cells. This feature allows us to assess the influence of transgene expression in Purkinje cells by comparing the morphology and electrophysiology of transduced Purkinje cells with those of neighboring non-transduced Purkinje cells from the same animal.

To identify a transgenic founder line that expresses sufficient levels of a transgene under the control of the Purkinje cell-specific L7 promoter, cerebellar sections of the F1 offspring should be examined. In our MSCV promoter model, we can predict the transgene expression profile in the founder mouse cerebella without euthanizing the F1 animals by examining the frequency of transduced circulating leukocytes in the founder mice. If a fluorescent protein, such as GFP, is co-expressed with a gene of interest under the control of the MSCV promoter, the strength of the peripheral fluorescence signal can be used as an index of the expression level of the gene of interest in the cerebellum. Thus, these peripheral indicators may help reduce the amount of time required to select founder lines with sufficient cerebellar transgene expression. Further studies are warranted regarding the superiority of the MSCV promoter for the generation of transgenic animals. Due to its unique features, the MSCV promoter holds great promise for future basic and translational research.

## Materials and Methods

All procedures for the care and treatment of animals were performed according to the Japanese Act on the Welfare and Management of Animals and the Guidelines for the Proper Conduct of Animal Experiments issued by the Science Council of Japan. The experimental protocol was approved by the Institutional Committee of Gunma University (09-062 and 09-051). All efforts were made to minimize suffering and to reduce the number of animals used.

### Virus Preparation

HIV-1-based self-inactivating lentiviral vectors obtained using pCL20c plasmids [11], [12] were used for the generation of transgenic mice. Vesicular stomatitis virus–G protein (VSV-G)-pseudotyped lentiviral vectors were produced as previously described [17]. Viral titers were assessed by counting the number of GFP-expressing cells after transduction in human embryonic kidney (HEK) 293T cells, as previously described [17].

### Dissociated Cerebellar Cultures and Infection with Lentiviral Vectors

Dissociated neuronal cultures from postnatal day 0 mouse pups were prepared as previously described [25]. Infection of dissociated cerebellar cultures in 12-well plates was conducted by replacing the culture medium with medium containing the virus [17].

### Immunocytochemistry

The dissociated cultures were fixed with 4% paraformaldehyde in phosphate-buffered saline (PBS)(–) for 30 min at room temperature, washed three times with PBS(–), then permeabilized and blocked with PBS(–) containing 0.2% Triton X-100, 2% normal goat serum and 2% bovine serum albumin for 30 min at room temperature. The cultures were then incubated with a mouse monoclonal anti-neuron-specific nuclear protein antibody (NeuN; MAB377; Millipore, Billerica, MA, USA) at a concentration of 1∶500 for 2 h at room temperature. Bound primary antibody was detected with Alexa Fluor 568-conjugated goat anti-mouse IgG (Invitrogen, Carlsbad, CA, USA).

### Generation of Transgenic Mice

C57BL/6J or ICR females purchased from Japan SLC were super-ovulated by intraperitoneal injection of serotropin (5 IU; ASKA Pharmaceutical Co., Tokyo, Japan) followed 48 h later by gonatropin (5 IU; ASKA Pharmaceutical Co.) and were then allowed to mate with males of the same strains. Two-cell embryos were collected from the oviducts of the mated females 41 h after the gonatropin injection. The embryos were incubated at 37°C with 5% CO_2_ for 2 h prior to lentiviral injection. Two-cell embryos were injected with lentiviral vectors using an Eppendorf FemtoJet microinjector (Eppendorf AG, Hamburg, Germany) and a three-axis hanging joystick oil hydraulic micromanipulator (Narishige, Tokyo, Japan). The embryos were placed in 0.25 M sucrose in PB1 medium to expand the perivitelline space. The lentiviral solution was then injected into the perivitelline space. After injection, the embryos were incubated in mW medium (Mitsubishi Chemical Medience, Tokyo, Japan) for 1 h and were subsequently transferred into the oviducts of pseudo-pregnant females. For genotyping, genomic DNA extracted from the toe tips of weaned pups was subjected to PCR amplification to determine the presence of the EGFP transgene using the following primers: EGFP-F (AGCACATGAAGCAGCACGACTTCTT) and EGFP-R (AGGAACTCCAGCAGGACCATGTGAT). F1 transgenic mice were obtained by crossing a founder male mouse showing bright GFP body fluorescence (IC0Tg-6, see Table 1) to wild-type females. All 15 F1 mice we obtained carried a transgene likely due to insertion of transgenes at multiple loci in the genome of the parent transgenic mouse. Since there was no genotype-negative wild type in the F0 generation and also in the F1 offspring, we purchased 3 wild-type ICR mice as a control (IAWt-1, IAWt-2 and IBWt-3 in Table 1). In the C57BL/6 mouse study, as we obtained wild-type animals together with the transgenic mice, the wild-type littermates were used as a control.

### Flow Cytometry

Peripheral blood cells were collected from the tail veins of 12 MSCV-GFP founder mice using heparin-PBS. After removing the red blood cells by hemolysis with ACK lysis buffer, the cells were stained with anti-mouse CD45-PE mAb (clone 30-F11, eBioscience, San Diego, CA). Whole leukocytes were analyzed using a FACSCalibur flow cytometer and CellQuest Pro software (Becton Dickinson).

### Histological Analysis and Fluorescent Imaging

Mice were deeply anesthetized with sodium pentobarbital (40 mg/kg body weight) and were then transcardially perfused with a fixative containing 4% paraformaldehyde in 0.1 M phosphate buffer. Fluorescent images of the brains and bodies of the mice were obtained using a CCD camera (VB-7010; Keyence, Osaka, Japan) attached to a fluorescence stereoscopic microscope (VB-L10; Keyence). The brains were cut into 50 µm sagittal sections using a microslicer (DTK-1000; Dosaka, Kyoto, Japan). GFP fluorescent images were obtained using a fluorescence microscope (DMI6000 B; Leica, Nusslock, Germany) or a confocal laser-scanning microscope (LSM 5 PASCAL; Carl Zeiss, Oberkochen, Germany).

For quantifying the ratio of the number of transduced Purkinje cells to the total number of Purkinje cells, a cerebellar cortical image centered around lobule 6 was obtained at 100× magnification from sagittal sections that were immunolabeled for calbindin. The ratio of the number of transduced Purkinje cells to the total number of Purkinje cells was determined by counting the number of GFP-positive Purkinje cells in a group of 100 Purkinje cells. Bergmann glia, whose soma are localized in the Purkinje cell layer, were discriminated by their unique morphology; Purkinje cells have a large, approximately 20 µm diameter soma with well-differentiated dendrites, while Bergmann glia have a much smaller soma of approximately 10 µm in diameter from which a radial process extends to the cerebellar surface.

### Immunohistochemistry

Tissue sections were blocked with PBS containing 2% bovine serum albumin, 2% normal goat serum and 0.4% Triton X-100 for 1 day. Sections from the MSCV-GFP mice were treated with an affinity-purified mouse monoclonal anti-CD45 (eBioscience) or mouse monoclonal anti-a-actinin (Sigma-Aldrich, St. Louis, MO, USA) antibody at a 1∶500 dilution for 1 day at 4°C. Bound primary antibodies were detected with Alexa Fluor 568-conjugated goat anti-rat IgG (Invitrogen) for CD45 or Alexa Fluor 568-conjugated goat anti-mouse IgG (Invitrogen) for a-actinin. For Figure S1B-D, cerebellar sections were double immunostained with rat monoclonal anti-GFP (1∶1,000; clone GF090R, Nacalei Tesque, Kyoto, Japan) and mouse monoclonal anti-Calbindin D-28k (1∶500; Swant, Bellinzona, Switzerland), mouse monoclonal anti-S100 antibody (1∶1,000; Sigma) or rabbit polyclonal anti-Factor VIII-related antigen (1∶10; Biomeda, Foster, CA, USA). Bound primary antibodies for GFP, calbindin D-28k, S100 and factor VIII-related antigen were visualized with Alexa Fluor 568-conjugated donkey anti-rat IgG (1∶1,000) (GFP), Alexa Fluor 568-conjugated donkey anti-mouse IgG (1∶1,000; Invitrogen) (calbindin D-28k and S100) or Alexa Fluor 568 donkey anti-rabbit IgG (1∶1,000; Invitrogen) (factor VIII-related antigen). Fluorescent images of the cerebellar slices were obtained using a confocal laser-scanning microscope (LSM 5 PASCAL; Carl Zeiss).

### Southern Blot Analysis

For genomic DNA extraction, tails from the C57BL/6 MSCV-GFP founder (F0) mice and 15 offspring (F1) or non-transgenic mice (WT) were incubated in lysis buffer with proteinase K (Qiagen, Hilden, Germany). Five micrograms of DNA were digested overnight at 37°C with *Bam*HI (10 IU). *Bam*HI-digested DNA was separated by electrophoresis on a 0.8% agarose gel and was transferred to a Hybond N+ nylon membrane (GE Healthcare, Piscataway, NJ, USA). Bound DNA was hybridized with a DIG-labeled probe at 65°C (Fig. 4H). The probe was designed to target a 450 bp region that included the boundary between the MSCV promoter and GFP (Fig. 4H) and was synthesized from the pCL20c MSCV-GFP plasmid by PCR using the specific primers 5′-CGGATCGCTCACAACCAGTC-3′ (forward) and 5′-CACCACCCCGGTGAACAG-3′ (reverse) according to the protocol in the DIG DNA Labeling Kit (Roche Diagnostics, Penzberg, Germany). The hybridized probe was detected with an alkaline phosphatase-conjugated anti-DIG antibody (diluted 1∶10,000; Roche Diagnostics) and was visualized using a chemiluminescence substrate (CSPD, Roche Diagnostics). One *Bam*HI site was localized in the region hybridized with the probe (Fig. 4H), which divided the probe-target region into 2 segments (393 bp and 57 bp). However, because more than 100 bp is needed for hybridization with a probe at 65°C [26], the probe was expected to hybridize only with the 5′-side fragment containing the 393 bp probe-complementary sequence.

## Supporting Information

Figure S1 **GFP expression profile in the cerebellar cortices of 12 MSCV-GFP founders.** (A) Native GFP fluorescent images of sagittal sections of the cerebellar vermis. (B–D) Examples of different GFP expression patterns in the MSCV-GFP founders. Cerebellar sections from MSCV-GFP founder mice were double immunolabeled for GFP and a marker for Purkinje cells, that for Bergmann glia or that for endothelial cells. Immunolabeling for calbindin, a marker for Purkinje cells, showed Purkinje cell-specific GFP expression (B). In addition to Purkinje cells, some founders showed GFP expression in the Bergmann glia, which were immunolabeled by S100 (arrowhead, B), or in endothelial cells, which were immunolabeled by factor VIII-related antigen (arrowhead, D). The ID in each image corresponds to that in Table 1. Scale bar, 20 µm.
(TIF)

Figure S2 **GFP expression in the pontine nuclei of a C57BL/6 MSCV-GFP mouse line.** (A–C) GFP expression in and around the pontine nuclei. Scale bars, 50 µm (A, B) and 20 µm (C).
(TIF)
