# Supplementary figures and images for: The Murine Stem Cell Virus Promoter Drives Correlated Transgene Expression in the Leukocytes and Cerebellar Purkinje Cells of Transgenic Mice
Source: PLoS One. 2012 Nov 30;7(11):e51015. doi: 10.1371/journal.pone.0051015 (PMC3511439; doi:10.1371/journal.pone.0051015)

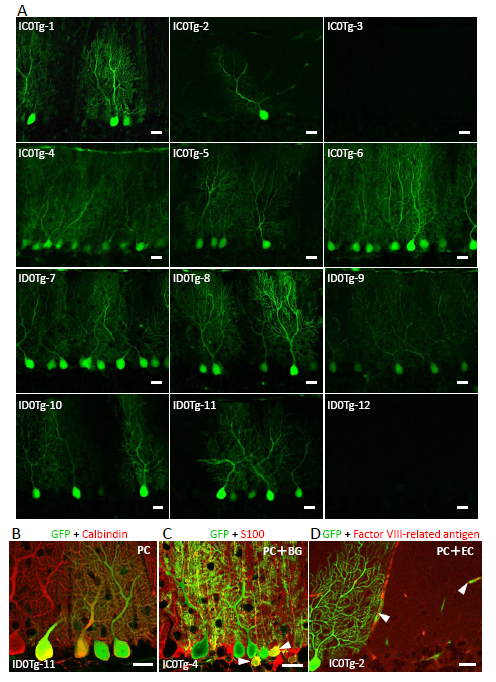

Supplement: Figure S1 — GFP expression profile in the cerebellar cortices of 12 MSCV-GFP founders. (A) Native GFP fluorescent images of sagittal sections of the cerebellar vermis. (B–D) Examples of different GFP expression patterns in the MSCV-GFP founders. Cerebellar sections from MSCV-GFP founder mice were double immunolabeled for GFP and a marker for Purkinje cells, that for Bergmann glia or that for endothelial cells. Immunolabeling for calbindin, a marker for Purkinje cells, showed Purkinje cell-specific GFP expression (B). In addition to Purkinje cells, some founders showed GFP expression in the Bergmann glia, which were immunolabeled by S100 (arrowhead, B), or in endothelial cells, which were immunolabeled by factor VIII-related antigen (arrowhead, D). The ID in each image corresponds to that in Table 1. Scale bar, 20 µm. (TIF) [file pone.0051015.s001.tif]

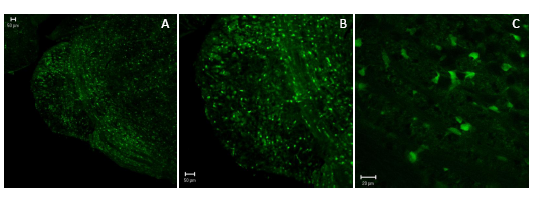

Supplement: Figure S2 — GFP expression in the pontine nuclei of a C57BL/6 MSCV-GFP mouse line. (A–C) GFP expression in and around the pontine nuclei. Scale bars, 50 µm (A, B) and 20 µm (C). (TIF) [file pone.0051015.s002.tif]
